# Disposable Voltammetric Sensor Modified with Block Copolymer-Dispersed Graphene for Simultaneous Determination of Dopamine and Ascorbic Acid in Ex Vivo Mouse Brain Tissue

**DOI:** 10.3390/bios11100368

**Published:** 2021-10-01

**Authors:** Dinakaran Thirumalai, Seulah Lee, Minho Kwon, Hyun-jong Paik, Jaewon Lee, Seung-Cheol Chang

**Affiliations:** 1 Department of Cogno-Mechatronics Engineering, College of Nanoscience and Nanotechnology, Pusan National University, Busan 46241, Korea; dinakaran@pusan.ac.kr; 2 College of Pharmacy, Pusan National University, Busan 46241, Korea; leeseulah@pusan.ac.kr (S.L.); neuron@pusan.ac.kr (J.L.); 3 Department of Polymer Science and Engineering, Pusan National University, Busan 46241, Korea; mhkwon89@pusan.ac.kr (M.K.); hpaik@pusan.ac.kr (H.-j.P.)

**Keywords:** dopamine, ascorbic acid, ex vivo brain tissue, Parkinson’s disease, reduced graphene oxide, block copolymer

## Abstract

Dopamine (DA) and ascorbic acid (AA) are two important biomarkers with similar oxidation potentials. To facilitate their simultaneous electrochemical detection, a new voltammetric sensor was developed by modifying a screen-printed carbon electrode (SPCE) with a newly synthesized block copolymer (poly(DMAEMA-*b*-styrene), PD*b*S) as a dispersant for reduced graphene oxide (rGO). The prepared PD*b*S–rGO and the modified SPCE were characterized using a range of physical and electrochemical techniques including Raman spectroscopy, scanning electron microscopy, transmission electron microscopy, cyclic voltammetry, electrochemical impedance spectroscopy, and linear sweep voltammetry. Compared to the bare SPCE, the PD*b*S–rGO-modified SPCE (PD*b*S–rGO/SPCE) showed better sensitivity and peak-to-peak separation for DA and AA in mixed solutions. Under the optimum conditions, the dynamic linear ranges for DA and AA were 0.1–300 and 10–1100 µM, and the detection limits were 0.134 and 0.88 µM (S/N = 3), respectively. Furthermore, PD*b*S–rGO/SPCE exhibited considerably enhanced anti-interference capability, high reproducibility, and storage stability for four weeks. The practical potential of the PD*b*S–rGO/SPCE sensor for measuring DA and AA was demonstrated using ex vivo brain tissues from a Parkinson’s disease mouse model and the control.

## 1. Introduction

Dopamine (DA) and ascorbic acid (AA) are important metabolites of significant biomedical interest. DA, a catecholamine neurotransmitter, is vital for regulating the central nervous system, including mood and motor function. Abnormal levels of DA can contribute to neurodegenerative diseases such as Parkinson’s disease (PD) and schizophrenia [1]. AA is an essential vitamin and prominent antioxidant, and it has been used to prevent or help treat the common cold, cancers, and mental illness [2]. AA plays an important role in synaptic formation and monoaminergic neurotransmission. In particular, it functions as a co-factor with dopamine-β-hydroxylase to donate electrons that convert DA to norepinephrine [3,4]. Furthermore, DA and AA coexist in the cerebrospinal fluid (CSF). Neuron’s uptake AA from the plasma through sodium-dependent vitamin C transporter-2 (SVCT2) and maintain a high internal AA concentration owing to its antioxidant property [5]. Therefore, the simultaneous determination of DA and AA is of immense interest in the fields of biochemistry and medicine. For such simultaneous determination of multiple metabolites, electrochemical sensors are ideal for mass production, on-spot rapid testing, and quality control assays for clinical diagnosis, owing to their excellent accuracy, selectivity, reproducibility, cost effectiveness, and ease of operation [6].

A major challenge in the simultaneous electrochemical detection of DA and AA is the overlapping electrooxidation peaks. Their very similar oxidative potentials result in a merged single peak [7]. Nanomaterials (including noble metals [8], various metal oxides [9,10], carbon-based nanomaterials [11,12], and polymers [13]) have been used to improve the catalytic selectivity of small molecules. Hence, they may also facilitate the simultaneous determination of DA and AA by simple and robust sensors.

Since its discovery in 2004, graphene, a single-carbon-atom-thick 2D macromolecule, has attracted substantial global attention [14]. Because of its unique properties, graphene is useful in many electrocatalytic applications. There has been extensive research on techniques of graphene synthesis, including mechanical exfoliation, chemical methods, and electrochemical approaches. However, these techniques suffer from several limitations, such as poor colloidal stability in various solvents and the agglomeration of graphene nanosheets [15].

Generally, graphene nanosheets are chemically reduced from graphene oxide (GO). When individual sheets form stable dispersions in organic and aqueous solutions, they could be conveniently combined with other materials to produce nanocomposites. Three major techniques, namely physical dispersion, covalent bonding, and noncovalent bonding could improve the homogeneous dispersion of graphene nanosheets. Among these, physical dispersion is relatively convenient, and the equipment is easy to operate. Nevertheless, this approach physically destroys the atomic arrangement, leaving defects that are difficult to rectify. Moreover, the dispersion rate and the proportion of graphene tend to be low. Therefore, the physical methods are often combined with other dispersion methods. Covalent bonding can be further classified as organic molecule bonding and polymer bonding, depending on the materials mixed into graphene. Although covalent bonding could improve the processability of graphene, it also causes significant physical defects and alters the chemical properties of graphene. Noncovalent bonding methods mainly employ π–π bonding, ionic bonding, and hydrogen bonding. These methods, conducted under mild conditions, are easy to use and introduce negligible defects in the atomic arrangement. However, noncovalent bonding may require additional components (such as surfactants) to improve the quality, quantity, and dispersibility of graphene in aqueous/non-aqueous media [16].

Certain polymers, surfactants, and aromatic molecules have been reported as dispersants to prevent the aggregation of graphene nanosheets. Block copolymers have shown better performance in this regard. Recently, Perumal et al. produced stable dispersions of graphene in ethanol, isopropanol, and methanol by employing polypyridine-, polystyrene-, and poly-*N*-vinyl-2-pyrrolidone-based block copolymers [17,18,19,20]. Qamar et al. reported a non-ionic block copolymer (Lugalvan BNO12) for dispersing graphene in aqueous solutions [21]. Our group also reported the dispersion of graphene in aqueous solutions using block copolymers such as poly(sodium 4-styrene sulfonate-*r*-LAHEMA) to prepare a sarcosine biosensor [22].

**Scheme 1:**
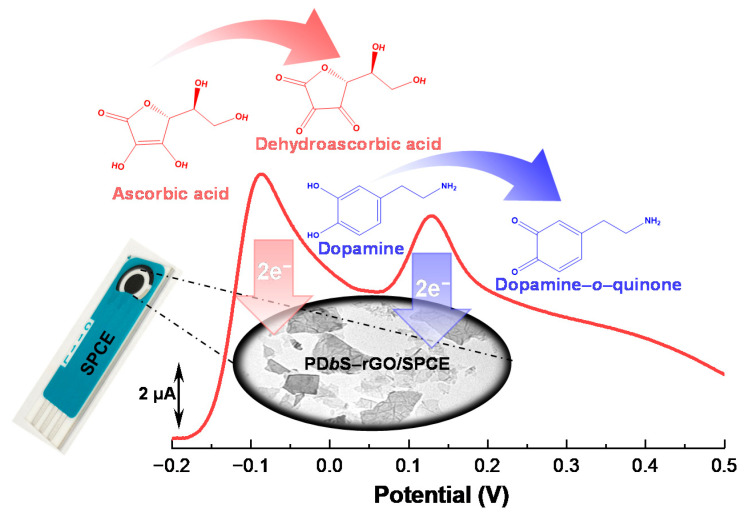
Schematic of the proposed PD*b*S–rGO/SPCE sensor for the simultaneous determination of AA and DA.

In this work, we use a newly synthesized block copolymer, poly(DMAEMA-*b*-styrene) (PD*b*S), as a dispersant for reduced graphene oxide (rGO) in aqueous media. The prepared PD*b*S–rGO is modified on the surface of a disposable screen-printed carbon electrode (SPCE) for the simultaneous electrochemical determination of DA and AA (Scheme 1). The experimental results suggest that the PD*b*S–rGO-modified SPCE (PD*b*S–rGO/SPCE) exhibits increased peak separation and improved electrocatalytic behavior toward the selective oxidation of DA and AA when compared to the rGO-modified and unmodified SPCE. The application prospect of PD*b*S–rGO/SPCE is demonstrated by simultaneously measuring the DA and AA concentrations in ex vivo brain tissue samples from both a 1-methyl-4-phenyl-1,2,5,6-tetrahydropyridine (MPTP)-induced PD mouse model and a control group.

## 2. Materials and Methods

### 2.1. Chemicals

GO was obtained from Nano Solution (Jeonju, Jeonbuk, South Korea). The following chemicals and reagents were purchased from Sigma-Aldrich (St. Louis, MO, USA): 2-cyano-2-propyl benzodithioate (CPBD), (2-dimethylaminoethyl)methacrylate (DMAEMA), styrene, azobisisobutyronitrile (AIBN), sodium phosphate monobasic (NaH_2_PO_4_), sodium phosphate dibasic (Na_2_HPO_4_), potassium ferricyanide (K_3_[Fe(CN)_6_]), AA, DA, uric acid, glucose, potassium chloride (KCl), MPTP, and anti-β-actin. Anti-tyrosine hydroxylase (TH) was purchased from Chemicon (Temecula, CA, USA). Phosphate buffer solution (PBS, 50 mM) was prepared following our previous report [23]. All other reagents were of reagent grade and used as received without additional purification. Triple distilled water (18 MΩ) obtained from a Milli-Q water purification system was used to prepare the solutions.

### 2.2. Synthesis of PolyDMAEMA Macro-RAFT Agent

In a typical reaction, AIBN (7.42 mg, 0.0452 mmol), CPBD (0.100 g, 0.452 mmol), and DMAEMA (45.7 mL, 0.271 mol) were dissolved in anisole (15.0 mL) in a 100-mL round bottom flask. After degassing the reactants with nitrogen for 30 min, the flask was immersed in a preheated oil bath at 90 °C under magnetic stirring. The polymerization was quenched by immersing the flask in iced water. The polymer was obtained by precipitation in cold hexane (twice) and then drying overnight in a vacuum drying oven.

### 2.3. Synthesis of Poly(DMAEMA-b-styrene) Copolymer

AIBN (3.44 mg, 2.09 × 10^−5^ mol), polyDMAEMA (PDMAEMA) macro-RAFT agent (9.00 g, 2.09 × 10^−4^ mol), and styrene (10.8 mL, 0.0941 mol) were dissolved in 15.0 mL anisole in a 100 mL round bottom flask. After degassing the reaction mixture with nitrogen for 30 min, the flask was placed in an oil bath preheated to 70 °C to initiate the polymerization reaction. The polymerization process was quenched by rapid cooling in iced water. The resulting PD*b*S polymer (Figure S1) dispersant was separated by precipitation in cold hexane and then vacuum drying overnight at room temperature.

### 2.4. Functionalization of rGO Using Polymer Dispersant

To functionalize rGO, approximately 500 mg of GO was dispersed in distilled water (500 mL) and placed in a 1000 mL round bottom flask to form a yellowish-brown dispersion. PD*b*S (5.00 g) was added to this dispersion under sonication for 1 h. To remove excess free polymer, the mixture was centrifuged at 33,800× *g* three times. Then, approximately 10 g of l-AA as a strong reducing agent was added to the PD*b*S-GO mixture, stirred for 1 h, and allowed to react at room temperature for 48 h. The black dispersion was sonicated for 30 min. This aqueous solution of PD*b*S-functionalized rGO (PD*b*S–rGO) showed good dispersion without any visible particulate matter. To remove the water-soluble AA and the pre-polymer, the dispersion was centrifuged three times. For the control experiments, an aggregation solution of rGO without PD*b*S functionalization was also prepared.

### 2.5. Preparation of PDbS–rGO-Modified SPCE

Screen-printed carbon electrodes (SPCEs, 4 mm diameter; model C11L) were purchased from Metrohm DropSens (Oviedo, Spain) and pretreated as described in our previous report [23]. Next, approximately 6 μL of PD*b*S–rGO was drop-cast on the SPCE surface and air-dried at room temperature for 30 min to obtain the PD*b*S–rGO/SPCE. Control rGO/SPCEs were prepared by similar steps using rGO aggregation solution without PD*b*S functionalization.

### 2.6. Instrument and Measurements

Field emission scanning electron microscopy (FE-SEM) images were acquired using a Zeiss GeminiSEM 500 (Carl Zeiss Microscopy Deutschland GmbH, Oberkochen, Germany). Transmission electron microscopy (TEM) images were obtained using a Hitachi H-7600 transmission electron microscope (Hitachi High-Tech. Corp., Tokyo, Japan). Raman spectra were recorded using an XperRam 200 (Nano Base, Seoul, South Korea) at a laser wavelength of 532 nm. Cyclic voltammetry (CV) and electrochemical impedance spectroscopy (EIS) measurements were conducted using an electrochemical workstation (model 604E; CH Instruments Inc., Austin, TX, USA). Linear sweep voltammetry (LSV) measurements were performed using a potentiostat (Compactstat, Ivium Technologies B.V., Eindhoven, The Netherlands).

The CV measurements were conducted in a disposable 2 mL electrochemical cell. First, the sensor was immersed in 2 mL of 0.1 M KCl containing 5 mM K_3_[Fe(CN)_6_], and the potential was then scanned from −0.2 to +0.6 V at various scan rates. EIS analysis was performed in the same solution in the frequency range of 100 kHz to 0.1 Hz at a formal potential of 250 mV and an AC amplitude of 5 mV. A 2 mL aliquot of 50 mM PBS containing DA and AA analytes (individually or mixed) was added to the cell. The analyte was pre-concentrated for 10 s at a potential of −0.2 V before CV and LSV. The cyclic voltammograms were recorded by potential scan between −0.2 and +0.5 V at the scan rate of 50 mV s^−1^, and the linear sweep voltammograms were recorded in the potential range of −0.2 to +0.5 V at a 10 mV s^−1^ scan rate. The resulting linear sweep voltammograms were baseline-corrected with the blank as a reference, and the extracted data are presented here.

### 2.7. MPTP-Induced PD Mouse Model

An MPTP-induced PD mouse model was used to explore ex vivo applications of the sensor. Male C57BL/6 mice (6 weeks old, 18–21 g) were obtained from Daehan Biolink Co., Ltd. (Chungbuk, South Korea). The animals were maintained under temperature- and light-controlled conditions (20–23 °C under a 12 h light and dark cycle) and provided with food and water *ad libitum*. The mice were randomly allocated to two groups (control and MPTP, *n* = 10) and acclimatized for one week before drug administration. MPTP was intraperitoneally injected at 20 mg/kg four times at 2 h intervals to induce acute PD symptoms. For the control group, the same volume of 0.1 M PBS (pH 7.0) was injected. The mice were sacrificed 24 h after injection, five mice in each group were used for histological analysis, and the striatum (STR) was collected from five other animals and used for sensor and biochemical analyses. The animal protocol used in this study was approved by the Pusan National University Institutional Animal Care Committee (PNU-IACUC; Approval Number PNU-2021-2961).

### 2.8. Immunohistochemistry

For histological analysis, the mice were intracardially perfused with 0.9% NaCl and fixed with 4% paraformaldehyde in 0.1 M PBS (pH 7.4). Cryoprotected brains were serially sectioned at 40 μm in the coronal plane using a freezing microtome (MICROM; Walldorf, Germany). The sections were immunostained for STR and substantia nigra (SN) using anti-tyrosine hydroxylase (TH; a dopaminergic neuronal marker) as previously described [24]. Images were acquired using a Nikon ECLIPSE TE 200-U microscope (Tokyo, Japan).

### 2.9. Western Blot Analysis

The brain tissues were homogenized, and the protein concentration was estimated using a BCA protein assay kit (Pierce, Shirley, NY, USA). The proteins (15 μg per lane) were separated by 12% sodium dodecyl sulfate-polyacrylamide gel electrophoresis (SDS-PAGE) and transferred to Immobilon-P^SQ^ membranes (Millipore; Burlington, MA, USA). The membranes were immediately placed in 5% non-fat milk for 30 min and then incubated with anti-TH and anti-β-actin antibodies overnight at 4 °C. Next, the membranes were washed and incubated with secondary antibody (Santa Cruz; CA, USA) for 2 h. Horseradish peroxidase-conjugated secondary antibody labeling was detected by enhanced chemiluminescence using a cooled CCD camera system (Atto Corp.; Tokyo, Japan).

### 2.10. Measurement of Reactive Oxygen Species (ROS)

Homogenized brain tissues STR regions (10 μL) were loaded in a black 96-well plate, and 50 mM phosphate buffer (PB) 190 μL) was added. The samples were then reacted with 25 μM dichlorofluorescin diacetate (DCF-DA) in PB, and the fluorescence intensities were measured at 5 min intervals using a GloMax fluorescence plate reader (Promega, Madison, WI, USA). 

### 2.11. Statistical Analysis

The significance of intergroup differences was determined by an unpaired *t*-test using Prism 7.0 (GraphPad Software Inc., San Diego, CA, USA). * *p* < 0.05, *** *p* < 0.001 were considered statistically significant.

## 3. Results and Discussion

### 3.1. Physical Characterization of PDbS–rGO

Raman spectroscopy is a powerful and non-destructive technique for characterizing the structure and electronic properties of functionalized rGO. In Figure S2a, the Raman spectra of rGO with and without PD*b*S functionalization both show typical D and G bands at approximately 1346 and 1587 cm^−1^, respectively, implying the presence of defective sites [25]. Notably, the intensity ratio of D to G (*I*_D_/*I*_G_) was higher for PD*b*S-functionalized rGO than that without (1.1 vs. 0.86). This slight increase in *I*_D_/*I*_G_ by PD*b*S functionalization may be due to additional structural defects formed during the functionalization process. Polymer functionalization not only creates defects but also modifies the electronic states of rGO. Therefore, the PD*b*S-functionalized rGO samples may contain more catalytic sites that help improve the sensor performance [26].

The surface morphologies of functionalized rGO were investigated using TEM and SEM. TEM images of the non-functionalized rGO showed aggregated multi-stacked nanosheet-like structures (Figure S2b). In contrast, the rGO sample dispersed in PD*b*S exhibited an even distribution of thin rGO films without any considerable aggregation, demonstrating that PD*b*S has a consistent dispersion capability (Figure S2c). The inset images show more aggregated rGO structures (Figure S2b inset) and uniformly dispersed rGO structures (Figure S2c inset).

**Figure 1:**
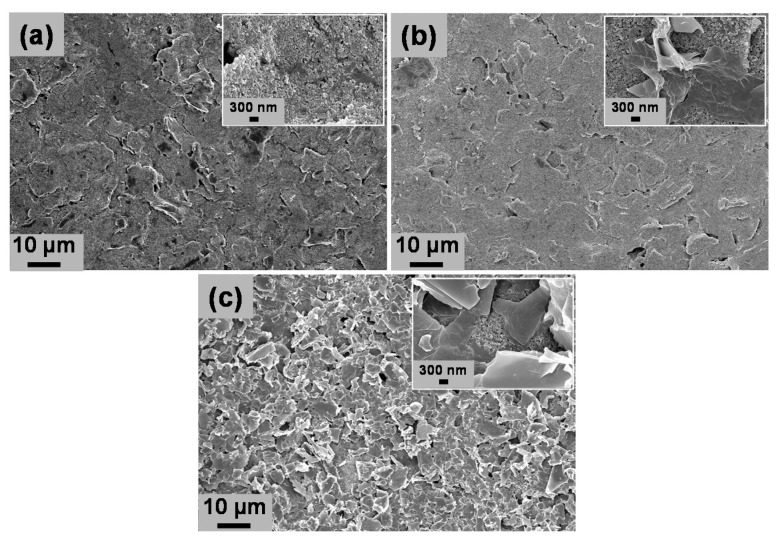
FE-SEM images of (**a**) bare SPCE; (**b**) rGO/SPCE; (**c**) PD*b*S–rGO/SPCE. Insets are the highly magnified images.

Figure 1 shows the SEM images of bare SPCE, rGO/SPCE, and PD*b*S−rGO/SPCE. rGO/SPCE had a slightly smoother surface than bare SPCE, and the magnified image (inset) shows multi-stacked nanosheet arrangements. PD*b*S−rGO/SPCE had a more wrinkled surface, and the magnified image displays evenly distributed rGO nanosheets without aggregation or layered morphology. These observations indicate that units of the block copolymer PD*b*S were distributed on the rGO nanosheets owing to the appropriate noncovalent interactions.

### 3.2. Electrochemical Characterization of Modified Electrodes

**Figure 2:**
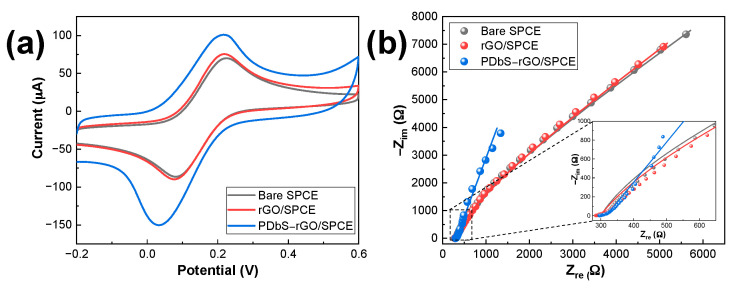
(**a**) CV curves for different SPCEs at a scan rate of 50 mV s^−1^; (**b**) Nyquist plots for different SPCEs in 5 mM K_3_[Fe(CN)_6_] prepared in 0.1 M KCl. The scattered dots in (**b**) represent the experimental data, and the lines are the theoretical fits.

The electrochemical behavior of the electrodes (bare SPCE, rGO/SPCE, and PD*b*S−rGO/SPCE) was characterized by CV and EIS using K_3_[Fe(CN)_6_] as a model redox probe. Figure 2a shows the cyclic voltammograms measured in a 5 mM K_3_[Fe(CN)_6_] solution containing a 0.1 M KCl supporting electrolyte at a sweep rate of 50 mV s^−1^. All electrodes exhibited well-defined oxidation and reduction peaks. The peak potential separation (Δ*E*p) for bare SPCE, rGO/SPCE, and PD*b*S−rGO/SPCE was 127, 128, and 172 mV, respectively. These values of Δ*E*p are greater than 57/*n* mV (*n* = number of electrons), the actual value for the reversible electron transfer reaction [27]. The current ratio between the anodic and cathodic peaks (*i*_pa_/*i*_pc_) for bare SPCE, rGO/SPCE, and PD*b*S−rGO/SPCE were 0.82, 0.85, and 0.67, respectively, which was less than 1, indicating quasi-reversible behavior. Therefore, all these characteristics suggest that the Fe^2+^/Fe^3+^ redox couple on the electrodes exhibits quasi-reversible behavior. In addition, the CV measurements were repeated at other scan rates (10–200 mV s^−1^) to investigate the electrochemical active surface area (ECSA) of the electrodes. Using the Randles–Ševčík Equation (1) and assuming a temperature of 25 °C [28]

(1) $$i_{p}=k\;n^{3/2}AD^{1/2}C\nu^{1/2}$$

where *i_p_* is the peak current (A), *k* is a constant 2.69 × 10^5^ C mol^−1^ V^−1/2^, *n* = 1 is the number of electrons involved in the electrochemical reaction, *A* is the electrode area (cm^2^), *D* is the diffusion coefficient of K_3_[Fe(CN)_6_] (7.20 × 10^−6^ cm^2^ s^−1^) [29], *C* is the analyte concentration (mol cm^−3^), and *ν* is the scan rate (V s^−1^). ECSA was calculated from the slope of the peak current (*i_p_*) vs. the square root of the scan rate (*ν*^1/2^). Compared with bare SPCE, modification with rGO hardly changed the ESCA (from 0.0840 to 0.0873 cm^2^), whereas the value of PD*b*S−rGO/SPCE was 41% higher (0.1325 cm^2^). Therefore, using PD*b*S as a dispersant for rGO clearly improved ECSA on the SPCE surface. It is worth noting that the much larger ECSA of PD*b*S−rGO/SPCE indicates that the rGO was uniformly dispersed with PD*b*S, which improved the surface-to-volume ratio of rGO.

EIS was used to compare the interfacial resistances of the electrodes, and the Nyquist plots of impedance are shown in Figure 2b. The results for bare SPCE and rGO/SPCE could be analyzed by the simple Randles circuit model that includes the solution resistance (*R*_S_), constant phase element (CPE), charge transfer resistance (*R*_CT_), and Warburg impedance (*Z*_W_). Analysis of PD*b*S−rGO/SPCE requires the addition of parallel capacitance (*C*_1_) and resistance (*R*_1_) to the simple Randles circuit model. The analysed data are listed in Table S1. Chi-square, the square of the standard deviation between the experimental data and the theoretical Randles fitting, was used to indicate the goodness of fit. The value of chi-square is less than 0.001 indicating that the model was a very good fit to the data. *n* is the exponent value (0 < *n* < 1), which is zero and one for an ideal resistor and an ideal capacitor, respectively. *R*_S_ and *Z*_W_ represent properties of the electrolyte solutions. *R*_CT_ and CPE are often affected by changes at the electrode/electrolyte interface. The *R*_CT_ values for bare SPCE, rGO/SPCE, and PD*b*S−rGO/SPCE were approximately 24.6, 29.5, and 11.4 kΩ cm^−2^, and the CPE values were approximately 57.1, 70.5, and 289.3 µF cm^−2^, respectively. These results demonstrate that PD*b*S−rGO/SPCE has better capability and accelerated kinetics for electron transfer.

**Figure 3:**
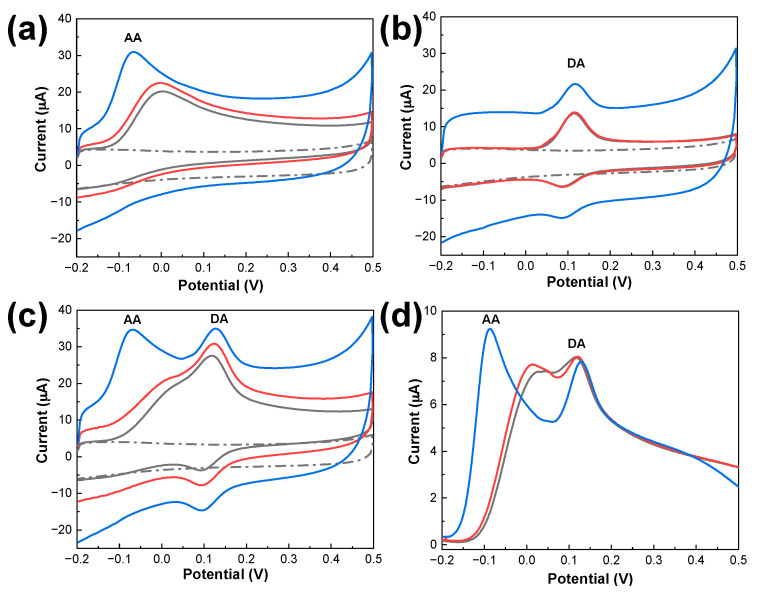
CV curves measured in (**a**) 500 µM AA; (**b**) 50 µM DA; (**c**) the mixture of 500 µM AA and 50 µM DA with bare SPCE (black line), rGO/SPCE (red line), and PD*b*S–rGO/SPCE (blue line). CV curves for bare SPCE measured in 50 mM PBS (pH 7.0) are represented as black dash–dot lines. Scan rate: 50 mV s^−1^; (**d**) LSV curves measured in a mixture of 500 µM AA and 50 µM DA for bare SPCE (black line), rGO/SPCE (red line), and PD*b*S–rGO/SPCE (blue line) at a scan rate of 10 mV s^−1^.

The electrocatalytic activity of the electrodes toward the oxidation of DA and AA was determined by CV measurements of each analyte. As illustrated in Figure 3a, a peak corresponding to the irreversible oxidation of AA was observed in bare SPCE, rGO/SPCE, and PD*b*S−rGO/SPCE. Compared with the anodic peak potential (*E*_pa_) of bare SPCE, the value for rGO/SPCE was slightly shifted toward the negative, from 3.81 to −1.78 mV, and that of PD*b*S−rGO/SPCE was further shifted to −65.3 mV. The *i*_pa_ values for rGO/SPCE and PD*b*S−rGO/SPCE were 5% and 22% higher than those of bare SPCE, respectively. The shift in *E*_pa_ and increase in *i*_pa_ can be attributed to the more abundant active sites and the higher surface-to-volume ratio of PD*b*S−rGO. In the case of DA (Figure 3b), a quasi-reversible voltammogram was obtained for bare SPCE, rGO/SPCE, and PD*b*S−rGO/SPCE with similar values of *E*_pa_ (near 115 mV) and *i*_pa_. The significant increase in charging current observed for PD*b*S−rGO/SPCE might be due to the double-layer capacitance of PD*b*S-dispersed rGO. 

The simultaneous determination of DA and AA was tested in a mixed solution containing 50 µM DA and 500 µM AA. As illustrated in Figure 3c, in the oxidative scan, bare SPCE (blank line) and rGO/SPCE (red line) showed only broad, single quasi-reversible voltammograms. These unresolved broad peaks signify that it is not feasible to detect DA and AA simultaneously using the two electrodes. In contrast, well-defined and independent oxidation peaks for DA and AA were observed for PD*b*S−rGO/SPCE (blue line in Figure 3c), indicating that PD*b*S−rGO/SPCE improved electrocatalytic activity for DA and AA oxidation.

We further studied the simultaneous determination of DA and AA with the bare SPCE, rGO/SPCE, and PD*b*S−rGO/SPCE using LSV (Figure 3d). In contrast to the CV curves, the LSV curves for bare SPCE (black line) and rGO/SPCE (red line) displayed two poorly separated oxidation peaks for AA and DA. In contrast, PD*b*S−rGO/SPCE (blue line) displayed well-defined separate oxidation peaks of AA and DA. The Δ*E*_p_ value of PD*b*S−rGO/SPCE (216.5 mV) is 2.8 times higher than that of bare SPCE (77.2 mV), and 2.1 times higher than that of rGO/SPCE (103.9 mV). These results further confirm that PD*b*S−rGO/SPCE can distinguish between the oxidation peaks of DA and AA. At pH 7.0, AA resumes its anionic form (p*K*_a_ = 4.1), while DA becomes cationic form (p*K*_a_ = 8.9). The PD*b*S dispersant containing DMAEMA compound has a known p*K*_a_ value of 8.4, and most of the DMAEMA molecules present in the PD*b*S are expected to exist as protonated species at pH 7.0 [30]. Because of its opposite electric charge, PD*b*S attracts and oxidizes AA first. PD*b*S might have no impact on similar charged DA. Therefore, the PD*b*S dispersant causes a shift in the AA oxidation peak but it has no impact on the DA oxidation peak. Furthermore, PD*b*S−rGO/SPCE demonstrated improved electrocatalytic activity for DA and AA oxidation. 

### 3.3. Optimization of pH

The effect of solution pH on PD*b*S−rGO/SPCE was studied over the pH range of 6.0–8.0. The results (Figure S3a) imply that the *i*_pa_ value of AA increases with increasing pH until pH 7.0, and then decreases at a higher pH. Similarly, the *i*_pa_ value of DA increases as the pH increases to a maximum at pH 7.0, followed by a drop to a mildly alkaline pH (Figure S3b). Figure S3c illustrates a linear relationship between *E*_pa_ and the solution pH. The Δ*E*_p_ values of the analytes are shifted negatively when the pH is increased (Figure S3d). Considering that a higher *i*_pa_ value and a sufficiently large Δ*E*_p_ were obtained for the DA–AA system at pH 7.0, and the need to mimic physiological conditions, we selected pH 7.0 as the optimum pH for the ensuing LSV experiments.

### 3.4. Electrochemical Response of PDbS−rGO/SPCE to DA and AA

**Figure 4:**
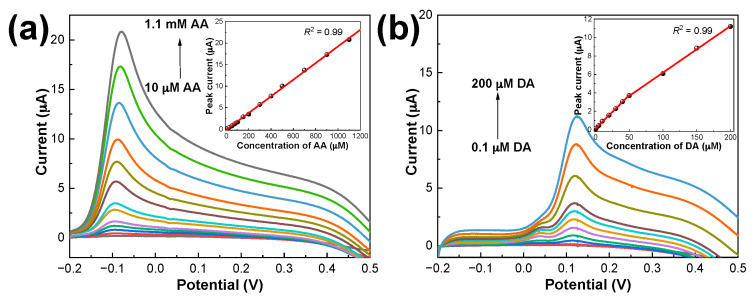
LSV curves for PD*b*S–rGO/SPCE in (**a**) 10 µM to 1.1 mM AA; (**b**) 0.1 µM to 200 µM DA prepared in 50 mM PBS (pH 7.0). Insets: Plots of anodic peak current against the concentrations of AA and DA, respectively.

The electrochemical oxidation behaviors of DA and AA on PD*b*S−rGO/SPCE were examined individually by LSV, and the results are shown in Figure 4. *i*_pa_ increased substantially as the DA and AA concentrations increased. In the AA concentration range from 10 µM to 1.1 mM, a sensitivity of 153.9 µA mM^−1^ cm^−2^ was achieved. Similarly, linear responses were observed for DA in two concentration ranges: from 100 nM to 50 µM (sensitivity: 578.3 µA mM^−1^ cm^−2^) and from 50 µM to 200 µM (sensitivity: 402.9 µA mM^−1^ cm^−2^). 

### 3.5. Simultaneous Determination of DA and AA Using PDbS−rGO/SPCE

Following the individual electrochemical oxidation of DA and AA, we attempted to simultaneously determine DA and AA in a single solution using LSV. Specifically, the concentration of one analyte (AA or DA) was fixed, while that of the other analyte was varied. The obtained LSV curves are plotted in Figure S4. When the solution contained 50 µM of DA and between 10 µM and 1.1 mM of AA, the experimental data (Figure S4c) demonstrated that the *i*_pa_ of AA remained proportional to the concentration of AA. Therefore, the presence of DA does not affect the determination of AA; at the same time, the AA peak current slightly alters the DA current responses. Similarly, AA had no effect on the determination of DA.

**Figure 5:**
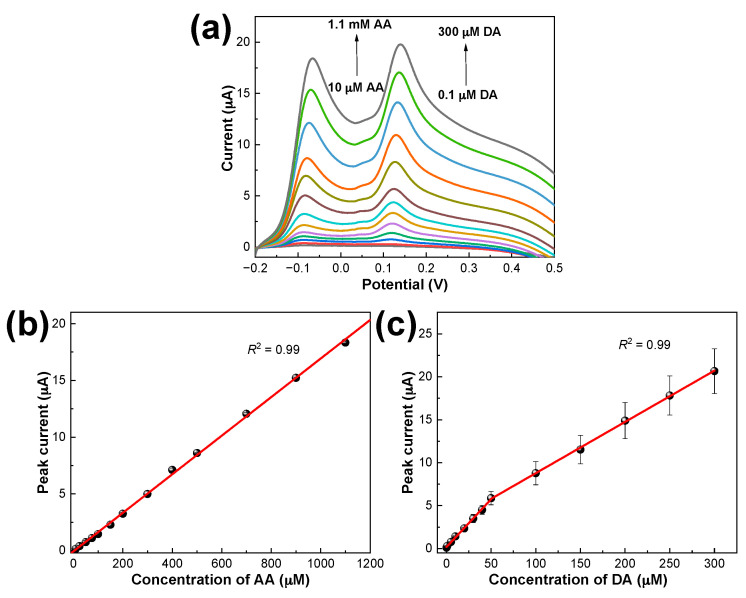
LSV curves of PD*b*S–rGO/SPCE in (**a**) 10 µM to 1.1 mM AA and 0.1 µM to 300 µM DA prepared in 50 mM PBS (pH 7.0); (**b**,**c**) Plots of the anodic peak current against the concentrations of AA and DA, respectively.

LSV curves were also collected for PD*b*S−rGO/SPCE while changing the concentrations of AA and DA simultaneously. Figure 5 shows that the two anodic peaks were well separated, and the *i*_pa_ of AA and DA increased proportionally with the corresponding analyte concentration. We calculated the limit of detection for PDbS–rGO/SPCE as 3σ/*m* where σ is the standard deviation of replicate blank and *m* is the slope from the calibration curves using standard approach 1 (SA1) [31]. Table 1 summarizes the analytical performance, including the linear range and limit of detection for the simultaneous determination of AA and DA using the proposed sensor, and compares the results with previous reports. The conclusion is that PD*b*S−rGO/SPCE could be applied for the simultaneous determination of AA and DA with a wide linear range and low detection limits.

### 3.6. Interference Study

The selectivity of PD*b*S−rGO/SPCE for determining DA and AA simultaneously in the presence of several interfering chemicals and ions (glucose, uric acid, Na^+^, K^+^, Mg^2+^, and NO^3−^) were studied. Even at a high concentration of 400 µM, these chemicals and ions had no noticeable effect on the current responses to DA (50 µM) and AA (200 µM) oxidation. However, uric acid produced an additional peak in the positive potential region, as shown in Figure S5. Our findings clearly demonstrate that these species, commonly found in biological samples, do not significantly interfere with the selective determination of DA and AA using PD*b*S−rGO/SPCE.

### 3.7. Reproducibility and Stability Studies

A mixed solution of 10 µM DA and 100 µM AA was used to assess the electrode-to-electrode reproducibility of five identically prepared PD*b*S−rGO/SPCEs. The relative standard deviations (RSDs) for DA and AA were calculated to be 5.0% and 5.1%, respectively. To test the stability of PD*b*S−rGO/SPCEs under ambient circumstances the electrodes were kept at 4 °C for four weeks and then used for the simultaneous determination of 100 µM AA and 10 µM DA. The LSV data revealed an obvious peak separation similar to the results in Figure 5, and the electrodes maintained 99.4% and 96.2% of the initial (i.e., freshly prepared electrodes) peak currents for AA and DA, respectively. These results indicate the excellent reproducibility and stability of PD*b*S−rGO/SPCE for the simultaneous detection of AA and DA.

### 3.8. Analysis of Brain Tissue Samples

**Figure 6:**
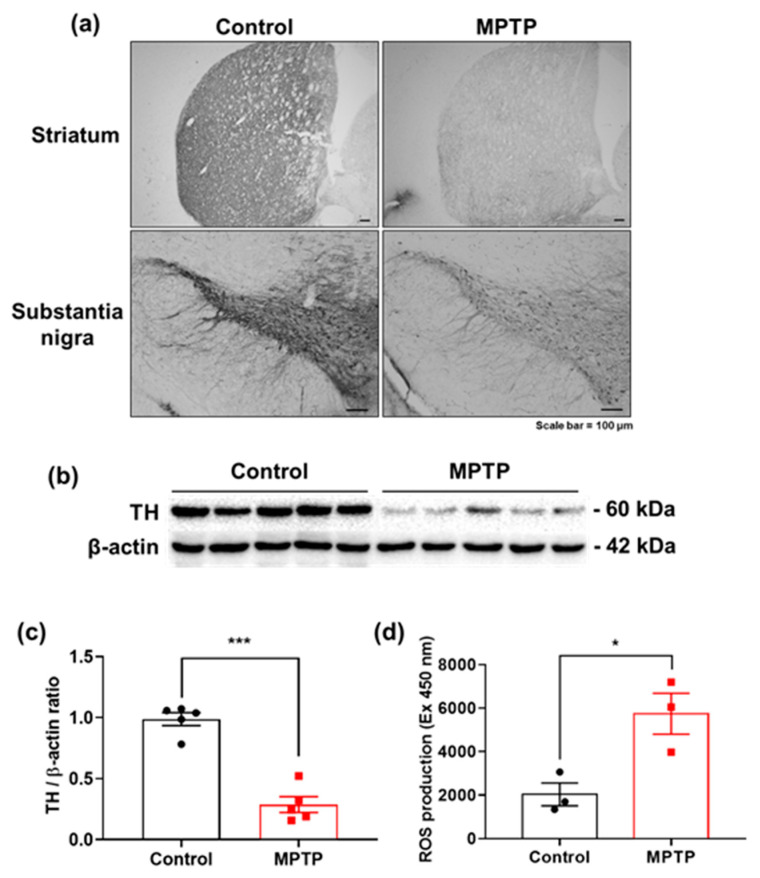
(**a**) Immunohistochemistry stained with TH antibody reveals that TH immunoreactivity clearly reduced by MPTP impaired the nigrostriatal pathway in both STR and SN. Scale bar = 100 µm; (**b**) Western blot analysis showing that TH levels in the STR region are lower in the MPTP-treated group than in controls; (**c**) Densitometric analysis of TH levels in the STR (control in black and MPTP in red color); (**d**) Level of reactive oxygen species (ROS) measured using DCF-DA, showing significantly elevated ROS production and increased oxidative stress in MPTP-treated STR. The results are presented as mean ± SE (*n* = 3–5 mice/group). * *p* < 0.05, *** *p* < 0.001 vs. naïve controls. The analysis was conducted by unpaired *t*-test with Fisher’s PLSD procedure (control in black and MPTP in red color).

To demonstrate the application potential of the prepared sensor for DA and AA determination in biological samples, the fabricated PD*b*S−rGO/SPCE was tested using the brain tissues of normal (control) and MPTP-induced PD mice by the standard addition method. MPTP is commonly used as a neurotoxin to mimic parkinsonism by inducing selective loss of dopaminergic neurons in the nigrostriatal pathway, ultimately leading to DA loss in the STR and SN [40]. The brain tissue samples were diluted 2× with 50 mM PBS (pH 7.0), and LSV measurements were performed under the optimum conditions. The measured AA and DA concentrations are summarized in Table S2. The concentrations of AA and DA in the PD samples were approximately 1.4 times lower than those in the control samples. The recovery percentages were less than 100%, possibly due to the matrix effect in the samples with complex composition [41,42]. Therefore, the sensor surfaces are typically protected by membranes, and further research on modification methods to improve the electrode performance may be required before applying the designed sensors to real-world samples. Our sensor results were validated by comparison with immunohistochemistry and Western blot analysis. The immunohistochemistry images clearly show that the DA levels in both STR and SN were reduced in MPTP-induced PD lesions (Figure 6a). Western blot analysis also confirmed significantly reduced DA levels in the STR (Figure 6b,c). More interestingly, the decreased AA and DA levels in the PD samples (Table S1) coincided with significantly elevated ROS levels (Figure 6d), suggesting that brain redox homeostasis was disrupted, and oxidative stress was increased by MPTP injection [43].

## 4. Conclusions

Block copolymer-dispersed rGO was successfully prepared and utilized to modify SPCE. The resultant PD*b*S−rGO/SPCE was applied for the separate and simultaneous electrochemical determination of DA and AA. PD*b*S–rGO/SPCE showed clear peak-to-peak separation for DA and AA, and it also demonstrated significantly improved electrocatalytic activity toward the oxidation of these two analytes. Moreover, the developed electrode exhibited the lowest detection limit compared with previous reports, and high selectivity, better reproducibility, and high stability. The sensor was successfully applied for ex vivo detection in the brain tissue of a PD mouse model, although the recovery was less than 100%. For use in biological samples with many complex interfering molecules, the electrode’s sensitivity may require improvement for the accurate measurement of DA and AA.

## Figures and Tables

**Table 1:** Performance comparison of proposed sensor with other reported sensors for simultaneous detection of DA and AA.

Electrodes	Dynamic Linear Range (µM)		Limit of Detection (µM)		Ref.
	DA	AA	DA	AA	
3D-MoS_2_/rGO/Au/GCE	0.30–198.3	2.0–5400	0.11	0.93	[32]
rGO/SPCE	0.20–80	100–2500	0.10	50	[33]
PEDOT/rGO/GCE	0.10–901	0.20–907	0.60	1.5	[34]
CM/AgNPs/SDS/MWCNTs/GCE	12–200	-	0.14	-	[35]
rGO/AgNWs/ AgNPs/SPCE	0.60–50	-	0.16	-	[36]
N-rGO/GCE	1.0–60	100–4000	0.10	9.6	[37]
Ti-C-Tx/GCE	0.50–50	100–1000	0.060	4.6	[38]
C_3_F_7_-azo+-TaWO_6_/GCE	50–310	50–310	0.81	1.6	[39]
PD*b*S–rGO/SPCE	0.10–300	10–1100	0.134	0.88	This work

3D-MoS_2_: 3D networked molybdenum disulfide; Au: gold nanoparticles; GCE: glassy carbon electrode; PEDOT: poly(3,4-ethylenedioxythiophene); CM: curcumin; AgNPs: silver nanoparticles; SDS: sodium dodecyl sulfate; MWCNTs: multi-walled carbon nanotubes; AgNWs: silver nanowires; N-rGO: nitrogen-doped reduced graphene oxide; Ti-C-Tx: titanium carbide MXene; C_3_F_7_-azo+: polyfluorinated azobenzene; TaWO_6_: tantalum tungstate.

## Data Availability

The data presented in this study are available on request from the corresponding author.

## Supplementary Materials

The following are available online at https://www.mdpi.com/article/10.3390/bios11100368/s1, Figure S1: Chemical structure of PD*b*S polymer; Figure S2: (**a**) Raman spectra of rGO/SPCE and PDbS–rGO/SPCE; TEM images of (**b**) rGO; and (**c**) PDbS–rGO. Insets: photographs of the corresponding samples, Figure S3: LSV curves of (**a**) PD*b*S–rGO/SPCE in 50 mM PBS containing 500 µM and 50 µM DA at pH = 6.0–8.0. Plots of (**b**) *i*_pa_ vs. pH; (**c**) *E*_pa_ vs. pH; and (**d**) Δ*E*_pa_ vs. pH., Figure S4: LSV curves of PD*b*S–rGO/SPCE in (**a**) 10 µM to 1.1 mM AA in the presence of 50 µM DA; (**b**) 0.1 µM to 200 µM DA in the presence of 200 µM AA; (**c**,**d**) Plots of the anodic peak current against the concentrations of AA and DA, respectively, Figure S5: Interference study of PD*b*S–rGO/SPCE in 200 µM AA and 50 µM DA with added compounds and ions: glucose, Na^+^, K^+^, Mg^2+^, NO^3−^, and uric acid (each at a concentration of 400 µM), Table S1: EIS parameters obtained by fitting the data to equivalent Randles circuit model of Figure 2b, Table S2: Simultaneous determination of AA and DA in mouse brain tissue samples using PD*b*S–rGO/SPCE.
