# Supplementary material for: Disposable Voltammetric Sensor Modified with Block Copolymer-Dispersed Graphene for Simultaneous Determination of Dopamine and Ascorbic Acid in Ex Vivo Mouse Brain Tissue
Source: Biosensors (Basel). 2021 Oct 1;11(10):368. doi: 10.3390/bios11100368 (PMC8534151; doi:10.3390/bios11100368)
Supplement: Supplementary file 1 [file biosensors-11-00368-s001.zip › biosensors-1393138-supplementary.pdf]

Supplementary Material

Disposable Voltammetric Sensor Modified with Block Copolymer-Dispersed Graphene for Simultaneous Determination of Dopamine and Ascorbic Acid in Ex Vivo Mouse Brain Tissue

Dinakaran Thirumalai [1,†], Seulah Lee [2,†], Minho Kwon [3,†], Hyun-jong Paik [3], Jaewon Lee [2] and Seung-Cheol Chang [1,*]

[1] Department of Cogno-Mechatronics Engineering, College of Nanoscience and Nanotechnology, Pusan National University, Busan 46241, Korea; dinakaran@pusan.ac.kr

[2] College of Pharmacy, Pusan National University, Busan 46241, Korea; leeseulah@pusan.ac.kr (S.L.); neuron@pusan.ac.kr (J.L.)

[3] Department of Polymer Science and Engineering, Pusan National University, Busan 46241, Korea; mhkwon89@pusan.ac.kr (M.K.); hpaik@pusan.ac.kr (H.-j.P.)

* Correspondence: s.c.chang@pusan.ac.kr

† These authors contributed equally to this work.

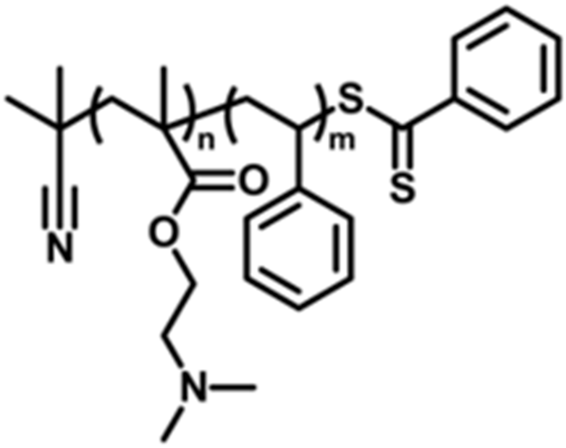

Figure S1. Chemical structure of PDbS polymer.

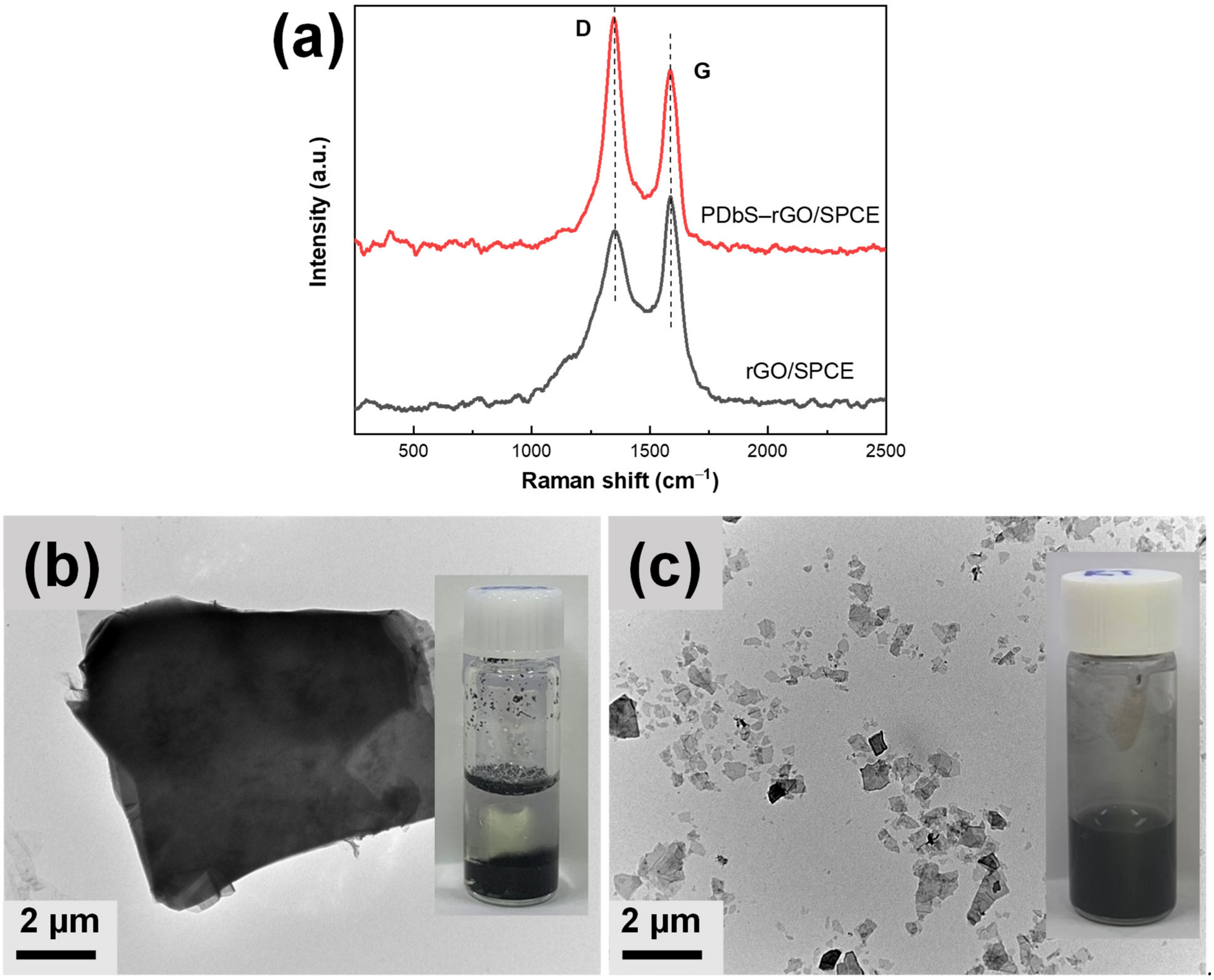

Figure S2. (**a**) Raman spectra of rGO/SPCE and PDbS–rGO/SPCE; TEM images of (**b**) rGO; and (**c**) PDbS–rGO. Insets: photographs of the corresponding samples.

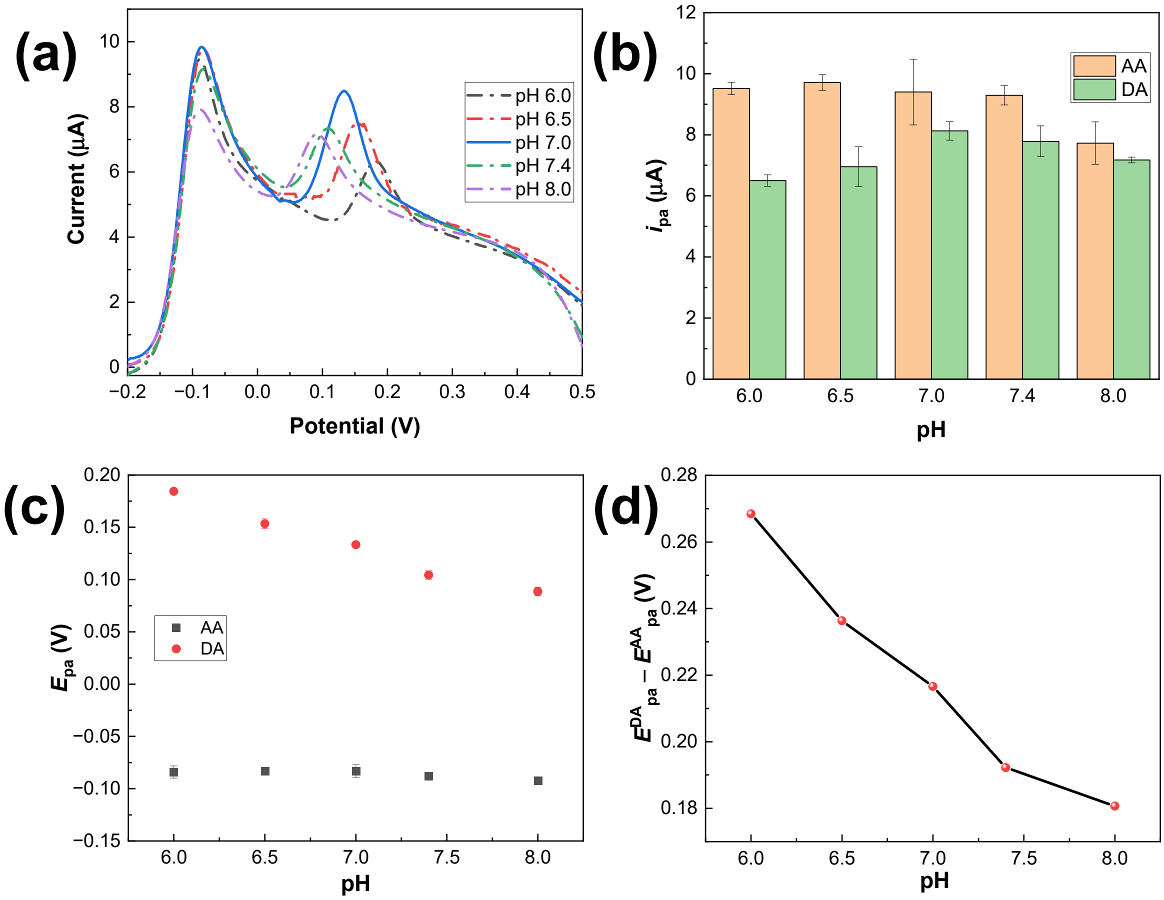

Figure S3. LSV curves of (**a**) PD*b*S–rGO/SPCE in 50 mM PBS containing 500 μM and 50 μM DA at pH = 6.0–8.0. Plots of (**b**) i_{pa} *vs*. pH; (**c**) E_{pa} *vs*. pH; and (**d**) ΔE_{pa} *vs*. pH.

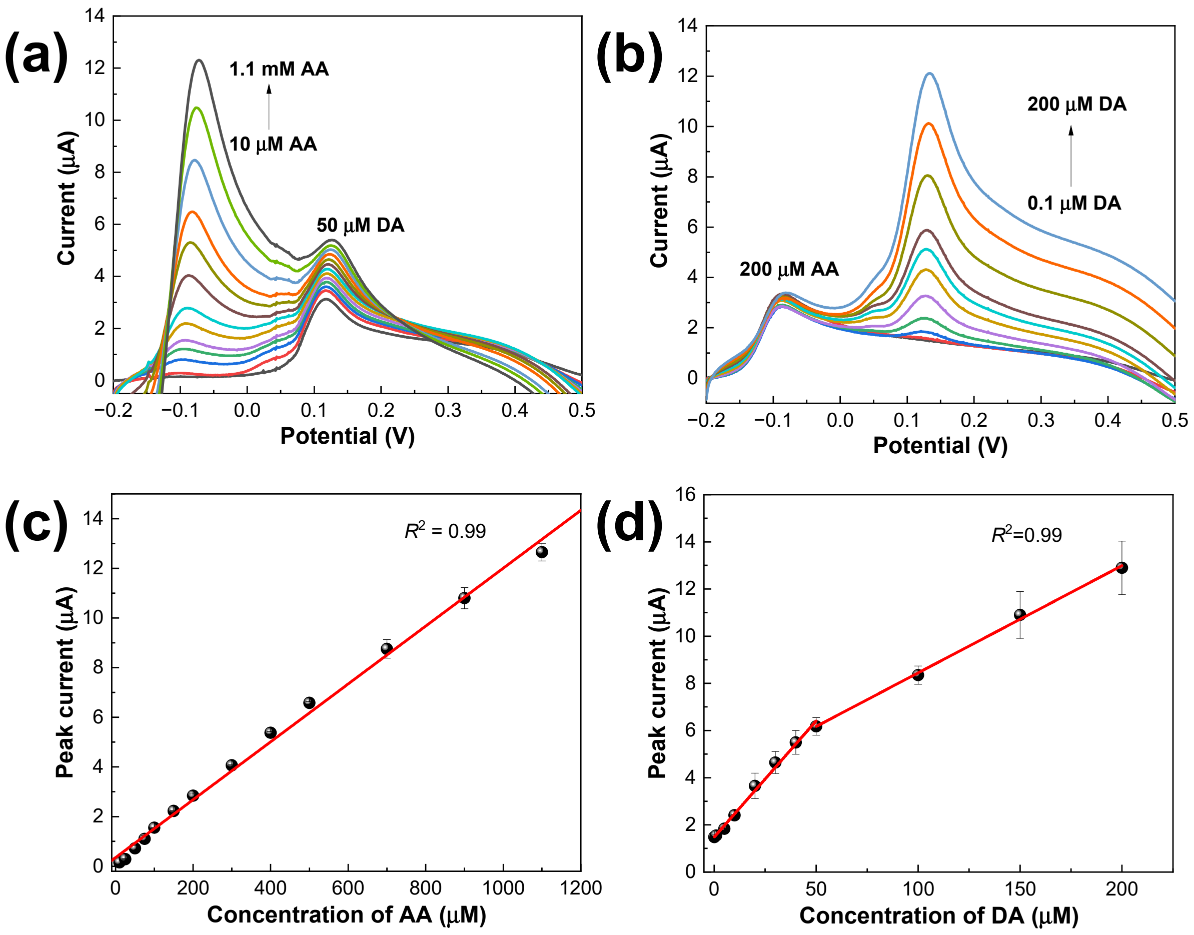

Figure S4. LSV curves of PD*b*S–rGO/SPCE in (**a**) 10 μM to 1.1 mM AA in the presence of 50 μM DA; (**b**) 0.1 μM to 200 μM DA in the presence of 200 μM AA; (**c, d**) Plots of the anodic peak current against the concentrations of AA and DA, respectively.

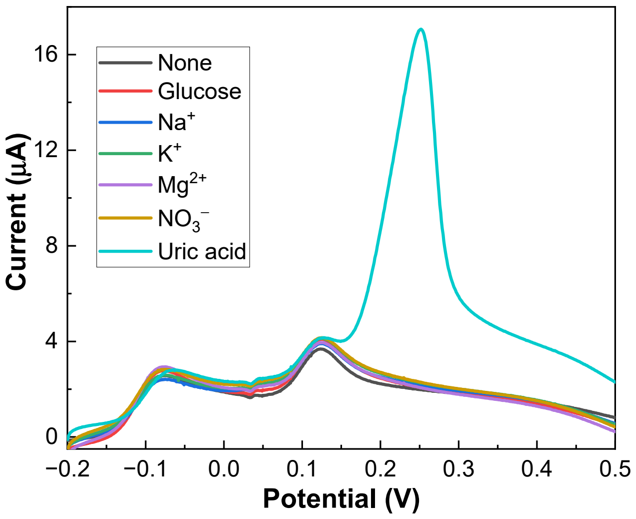

Figure S5. Interference study of PD*b*S–rGO/SPCE in 200 μM AA and 50 μM DA with added compounds and ions: glucose, Na^{+}, K^{+}, Mg^{2+}, NO^{3-}, and uric acid (each at a concentration of 400 μM).

Table S1. EIS parameters obtained by fitting the data to equivalent Randles circuit model of Figure 2b.

**Sensor**	**Chi-square**	***R*S (kΩ cm⁻²)**	***CPE* (μF cm⁻²)**	***R*CT (kΩ cm⁻²)**	***Z*W (kΩ cm⁻²)**	***n***	***C*1 (μF cm⁻²)**	***R*1 (Ω cm⁻²)**
Bare SPCE	0.000629	2.39	57.1	24.6	54.1	0.898	-	-
rGO/SPCE	0.00113	2.38	70.5	29.5	54.5	0.868	-	-
PD*b*S–rGO/SPCE	0.00103	2.38	289.3	11.4	23.4	0.853	30.8	137.7

Table S2. Simultaneous determination of AA and DA in mouse brain tissue samples using PD*b*S–rGO/SPCE.

Samples	Analyte	Added (μM)	Found (μM)	Recovery (%)	RSD (%)
Control	AA	-	**29.3 ± 2.1**	-	7.2
	AA	100	82.4 ± 2.3	63.7	2.9
	AA	200	134.4 ± 6.2	58.6	4.6
	AA	300	210.9 ± 12.1	64.0	5.7
	DA	-	**3.5 ± 0.03**	-	0.8
	DA	10	11.8 ± 0.2	87.4	1.9
	DA	20	19.9 ± 0.8	84.9	4.3
	DA	30	31.1 ± 0.8	92.7	2.7
PD	AA	-	**20.4 ± 2.4**	-	11.8
	AA	100	72.3 ± 10.0	60.1	13.8
	AA	200	123.8 ± 13.2	56.2	10.7
	AA	300	180.9 ± 21.5	56.5	11.9
	DA	-	**2.5 ± 0.3**	-	10.4
	DA	10	10.9 ± 1.3	87.3	11.5
	DA	20	20.3 ± 1.6	89.9	8.0
	DA	30	29.7 ± 2.1	91.4	7.1
